# Self-care in children and young people with complex chronic conditions: a qualitative study using Emotional Text Mining

**DOI:** 10.3389/fped.2023.1170268

**Published:** 2023-07-28

**Authors:** Giuseppina Spitaletta, Valentina Biagioli, Francesca Greco, Rachele Mascolo, Annachiara Liburdi, Giulia Manzi, Orsola Gawronski, Riccardo Ricci, Emanuela Tiozzo, Ercole Vellone, Teresa Grimaldi Capitello, Michele Salata, Massimiliano Raponi, Immacolata Dall’Oglio, Self-care CYP Study Group, Valentina Vanzi

**Affiliations:** ^1^ Professional Development, Continuing Education and Research Service, Bambino Gesù Children’s Hospital, IRCCS, Rome, Italy; ^2^ Department of Languages and Literatures, Communication, Education and Society, University of Udine, Udine, Italy; ^3^ Pediatric Semi-Intensive Care Area/Unit, Bambino Gesù Children’s Hospital, IRCCS, Rome, Italy; ^4^ Department of Biomedicine and Prevention, University of Rome “Tor Vergata”, Rome, Italy; ^5^ Unit of Clinical Psychology, Bambino Gesù Children’s Hospital, IRCCS, Rome, Italy; ^6^ Paediatric Palliative Care Unit, Bambino Gesù Children’s Hospital, IRCCS, Rome, Italy; ^7^ Medical Directorate, Bambino Gesù Children's Hospital, IRCCS, Rome, Italy; ^8^ Bambino Gesù Children's Hospital, IRCCS, Rome, Italy

**Keywords:** self care, self-management, chronic disease, pediatrics, adolescent, young adult, parents

## Abstract

**Objectives:**

To explore: (1) self-care behaviors in children and young people (range: 6 months–24 years) with complex chronic conditions, characterized by the diagnosis of a severe chronic condition, substantial family-identified needs, functional limitations associated with technology dependence, and intensive use of healthcare services; (2) the contribution to self-care of family members and other persons involved in the child's health and daily life context (e.g., health professionals and teachers), and (3) the principal factors that might have influenced the self-care process associated with developmental age.

**Methods:**

A qualitative descriptive study was conducted in an Italian academic tertiary pediatric hospital between September 2020 and May 2021. Overall, 25 focus groups and 7 online interviews were conducted via videoconferencing. Textual data were analyzed using Emotional Text Mining to identify three levels of communication: the factors, the main themes (clusters), and the sub-themes.

**Results:**

A total of 104 participants were enrolled, including 27 patients with complex chronic conditions (12 males, mean age = 11.1 ± 4.40), 33 parents, 6 siblings, 33 health professionals, and 5 teachers. Participants described the process of self-care through four main factors: “self-care”, “external settings”, “family”, and “management”. Five clusters (themes) were identified: (1) Self-care management (device; consulting); (2) Shift of agency (influencing factors; parents; school); (3) Self-care support (normal life and personal development; multidisciplinary support); (4) Daily self-care maintenance/monitoring; (5) Treatment adherence. Self-care management was mostly relevant for parents of children aged between 6 months and 3 years.

**Conclusion:**

The self-care process varies according to the needs related to the specific developmental age and the evolution of the clinical condition over time. The contribution of the family, health professionals, and social networks is fundamental for adequate self-care. To help families manage the unstable condition of their children at home, it is necessary to strengthen support networks implement home care, and ensure continuity of care.

## 1. Introduction

The number of children and young people with chronic conditions is increasing over time especially in upper-middle, and high income countries (1). In Italy, where the present study was conducted, children with chronic conditions account for between 10% and 18% of the pediatric population, and 1.6% suffer from two or more chronic conditions (2–4). Similarly, in the United States, the prevalence of any chronic health condition among children and young people is about 27% (5, 6) and approximately 17% have a special healthcare need (7). Moreover, approximately 0.4%–1.5% of all US children are affected by medical complexity (7, 8). According to Cohen et al. (9), the medical complexity in children is characterized by four domains: (a) family-identified healthcare service needs, (b) one or more chronic clinical condition(s), either diagnosed or unknown, (c) severe functional limitations, and (d) high projected utilization of health resources.

It is fundamental to actively involve children and young people and their families in managing their complex condition from the beginning of the health problem over time, and health professionals need to focus on child and family-centered care (10–12). In line with international guidelines, the Italian Chronic Diseases Plan aims to develop models that guarantee an integrative approach to meeting healthcare needs, especially at home, and that consider the developmental stage (13). In addition, encouraging family empowerment to cooperate synergistically with healthcare providers is considered fundamental to meeting children's needs, expectations, preferences, and values (14). Particular attention should be paid to the different development stages of children and young people with complex chronic conditions and their transition processes to adulthood. Indeed, specific needs might arise as children grow, especially when they become more involved in decision-making and develop their self-care skills (15).

Self-care is defined as “the ability of individuals, families, and communities to promote health, prevent disease, maintain health, and cope with illness and disability with or without the support of a health-care provider” (16). Riegel et al. (17) defined self-care in the adult population with chronic diseases as a process of maintaining health through health-promoting practices and illness management (17). Accordingly, self-care includes three core aspects: (a) self-care *maintenance*, behaviors used by individuals with chronic diseases to maintain physical and emotional stability; (b) self-care *monitoring,* the process of observing oneself for changes in signs and symptoms; (c) self-care *management*, the response to signs and symptoms when they occur, with the assessment of treatment efficacy (17).

Recently, Dall'Oglio et al. (18) designed a comprehensive model of self-care in children and young people with chronic conditions, by aggregating the main aspects of 13 conceptual models retrieved through a systematic review (18). This model regards self-care as a process based on health-promoting behaviours performed by children and young people with the support of the family during developmental age. Self-care behaviours include treatment adherence (e.g., taking all prescribed medications), healthy lifestyle (e.g., practicing sports or physical activity according to the child's possibilities and safely), symptom monitoring (e.g., monitoring how the child breathes), and response to symptoms (e.g., calling the healthcare providers when the situation becomes more complex). The self-care process is influenced by several factors and aims at improving health outcomes. Moreover, the shift of self-care agency from family to patients as the main actors of their self-care is emphasized. Since a description of behaviors that patients adopt to cope with their conditions is still missing, this model might be regarded as a starting point and a guide to inform care, assessing and promoting self-care.

Parent and family support (contribution to self-care) in daily activities of children and young people with chronic conditions are vital as they grow older. Family support improves treatment compliance and encourages motivation and willpower for care (19, 20). Parents might play a key role when acute and complex complications occur in their children since they might identify at a very early stage the predictive factors that require prompt actions (21). In addition, siblings can be successfully involved in contributing to self-care, even though they may be at an increased risk of depression due to their complex family situation (22).

In particular, health professionals could support self-care activities in children and young people with complex chronic conditions to promote their quality of life and improve their autonomy across developmental age, regardless of the complexity of their conditions (18, 23). In order to improve the quality of life of children and young people with complex chronic conditions, it is important to consider the deep influence that the condition has on the socialization experiences of these children and their families, which are vital for the child's healthy development (24, 25). Since children and young people are expected to deal with self-care activities at school, also teachers could contribute to facilitating social aspects related to self-care (26).

Since many chronic conditions are no longer considered a mortal threat, children and young people with specific disorders represent the future adult population (27). Self-care promotional approaches should start during childhood with the support of parents and be adapted to specific needs related to individual development (18). As such, self-care is fundamental to promote health over time despite the underlying disease. Although self-care has been largely explored in adults with chronic diseases (28–32), very few studies have explored self-care in children and young people (33, 34). In particular, no study has used Emotional Text Mining to analyze a large amount of data on self-care during developmental age, whereas this particular kind of sentiment analysis enables to identify elements from a large narrative text and social profiling to be performed (35–39).

Thus, the present study aimed to explore: (1) self-care behaviors in children and young people (age range: 6 months–24 years) with complex chronic conditions; (2) the contribution to self-care of family members (i.e., parents and siblings) and other persons involved in the child's health and daily life context (e.g., health professionals and schoolteachers); and (3) the key factors that might have influenced the self-care process associated with developmental age.

## 2. Methods

### 2.1. Design

This was a qualitative descriptive study using the Emotional Text Mining method for data analysis (39). The study was conducted at an Italian academic tertiary pediatric hospital between September 2020 and May 2021.

### 2.2. Participants

Participants were enrolled using judgment sampling, a non-probability purposive sampling technique whereby researchers select units to be sampled based on their professional judgment to collect data from the widest range of perspectives possible about a certain topic. The participants included inpatients and outpatients who were being cared for their chronic complex conditions by various departments of the hospital (e.g., the Department of Cardiac Surgery, Cardiology and Heart and Lung Transplantation; the Division of General and Specialized Pediatrics; and the Department of Surgery). To explore the process of self-care, focus groups and one-to-one interviews were conducted with patients, parents, siblings, health professionals, and schoolteachers via videoconferencing. We used this remote approach due to social distancing for the COVID-19 pandemic. A web conferencing platform was chosen as it was expected to be an effective strategy to overcome the challenges that occurred during the COVID-19 pandemic, offering advantages similar to those of face-to-face focus groups (40).

Participants were enrolled in the study if they met the following inclusion criteria: (a) children and young people up to 24 years old with a complex chronic condition, as described by Cohen et al. (9), (b) children and young people with a diagnosis of complex chronic condition for least six months since the study was conducted, (c) parents or siblings caring for children and young people, (d) health professionals caring for patient participants in hospitals and/or in the community for at least one year, and (e) other individuals who are part of the social life of children and young people, such as schoolteachers. The exclusion criteria were: (a) children and young people living with non-complex chronic conditions or with cancer, (b) children and young people up to 24 years old with mental and/or neurodevelopmental disorders as reported in the DSM-5 classification system (41), (c) patients, parents or other caregivers who could not speak Italian, (d) patients in critical conditions or with an advanced stage disease, and (e) abandoned patients or in foster care.

Our study sample included several groups of participants. To achieve maximum representation of each category, the population was divided according to the type of participant and age group. Six age groups were established for indirectly enrolled children and young people with complex chronic conditions: 6 months–2 years; 3–5 years; 6–10 years; 11–13 years; 14–17 years; and 18–24 years. We aimed to enroll parents for each age group and patients per each age group starting from the age of 6 years to 24 years. Siblings were grouped into two age groups: 6–15 year-old and 16–24 year-old group. Moreover, we enrolled other persons involved in the child's health and daily life context, such as healthcare professionals (nurses, pediatricians of the hospital, family pediatricians, psychologists, and physiotherapists) and schoolteachers. Overall, we aimed to include a total of 120 participants.

### 2.3. Data collection

Data were collected through focus groups. Focus groups were scheduled according to the type of participants (e.g., parents), age group (e.g., 14–17 years of age), and participants' availability. Each focus group aimed to include at least five participants but in case they were unavailable at least three participants were required. Moreover, some individual interviews were conducted instead of focus groups, to facilitate the active participation of younger patients or siblings (e.g., 6–10 years old) or to collect experiences of some parents who could not participate at the same time as the others. The integration of focus groups and individual interviews avoided the loss of important data and increased data richness (42). Nevertheless, this approach helped to explore the topic of the study from different perspectives. This methodology has been successfully used also in other studies (43, 44).

Moreover, socio-demographic and clinical data were collected for patients (e.g., age, gender, education level, diagnosis, the complexity of the disease and treatment), parents (e.g., age, gender, family components, educational level, and occupation), and siblings (e.g., age, gender, and education level). Socio-demographic and information about their job was collected from health professionals (e.g., age, gender, profession, educational level, the clinical setting where they worked, and years of work experience within the current clinical setting) and for schoolteachers (e.g., age, gender, educational level, years of work experience within the child's context of life). These data were collected online, after consent had been provided.

Focus groups and interviews were conducted using the Zoom Cloud Meetings platform, a video-conferencing service. The focus groups, using the audio and video options provided by the platform, allowed the participants to synchronously join the discussion, answer the questions, and comment in real-time. The web conferencing tool enabled to participate safely and freely in discussions using various mobile devices (tablets, laptops, smartphones). Technical support was provided to the participants to ensure that focus groups and interviews were conducted without interruptions.

Each focus group was led by a moderator who asked questions, while an observer took notes. The interviews were conducted by a moderator, the observer had the webcam switched off. An interview guide with three open-ended questions was used for all the focus groups and individual interviews (Supplementary Table S1). The questions explored the three core aspects of self-care according to the theory by Riegel et al. (17), in line with the comprehensive model of self-care in children and young people with chronic conditions (18). The questions were modified and simplified, as needed, so they could be easily understoon by the various types of participants. The moderator kept an open attitude during the focus groups/interviews, by asking probing questions when participants were silent and paraphrasing what participants expressed during the focus groups/interviews.

### 2.4. Ethical considerations

The Ethics Committee of the hospital where the study was conducted approved this study [2200_OPBG_2020]. All participants were informed about the purpose and procedures of this study and were asked to sign an informed consent form. The Ethics Committee had approved the option of online consent using a specific survey software in a video call with a research assistant. Parents were informed of the study objectives and were asked to provide a written informed consent. Data were collected anonymously using an identification code.

### 2.5. Data analysis

Participants' socio-demographic and clinical data were analyzed using descriptive statistics, including frequencies and percentages for categorical variables, and means (M) and standard deviations (SD) for continuous variables. The interviews and focus groups were recorded, and the content was transcribed verbatim, using an automatic online transcription system. Transcripts were merged and analyzed to identify three levels of communication: the factors, the main themes (clusters), and the sub-themes (45, 46), using the Emotional Text Mining method (39, 45). This is based on an automatic, bottom-up approach to natural language processing (a method that can accurately extract information and insights contained in the documents), allowing to analyze a large amount of textual data (39). It identifies both the semiotic (signs—the symbolic matrix) and the semantic (meanings—the word co-occurrence) level of communication. Emotional Text Mining performs a sequence of synthesis procedures, from preprocessing of merged transcripts (corpus) to term selection (keywords), clustering, and factorial analysis (47). It was used in different domains, including healthcare (35–38, 47).

In particular, we cleaned and preprocessed the textual data of the transcripts. To assess whether it was possible to statistically process data, we calculated the main lexical indexes: the total number of words (token), the number of different words (types), the ratio of the number of words occurring only once to the total number of different words (hapax percentage), and type/token ratio (48).

Words were lemmatized (grouped together according to their meaningful base form—lemma) using T-Lab dictionary (49), thus reducing the overall number of terms. We selected the terms of medium rank frequency, by filtering out the words in the high and low rank of frequency (45). All the documents of the merged transcripts were subdivided into context units (pieces of text). In order to detect the associative links between the words (the representations), we performed a cluster analysis with a bisecting *k*-means algorithm based on cosine similarity (50) on the term-context units matrix. The analysis was limited to twenty partitions, excluding all the text that did not have at least two keywords co-occurrence to classify the text. We calculated the intraclass correlation coefficient (*ρ*) index and evaluated the dendrogram to choose the optimal solution (number of clusters). The terms co-occurring in the cluster were also interpreted. Context units classified in each cluster were ordered according to their relevance (score) (49). The reading of the most representative context units of each cluster made it possible to define more thoroughly the judges' interpretations. Results were compared among judges and a final agreement was found (45). In order to detect the symbolic matrix, we performed a correspondence analysis on the term-cluster matrix (51). Four judges first interpreted separately the factorial space to identify the factors that represented the general axes of communication. Moreover, the cluster analysis results were used to identify the main themes (clusters) and sub-themes, according to the location of the clusters in the factorial space.

Finally, to assess participants' themes specificities (differences among participants), we performed a chi-square test on the cluster-type of the participants’ contingency table for the entire sample and a second chi-square test on the cluster-patients' age group contingency table for patients, parents, siblings separately, using the standardized residuals to identify important differences (52).

### 2.6. Validity and reliability

Two researchers conducted verification and any modification, or integration of the transcripts generated by the automatic online transcription system. Emotional Text Mining was employed for data analysis by an expert researcher (FG) together with other three researchers who confirmed each step. Overall, four researchers contributed to the interpretation of the factorial space and the cluster analysis results.

## 3. Findings

### 3.1. Characteristics of the participants

Overall, 104 participants were interviewed including 27 patients, 33 parents, 6 siblings, 33 healthcare professionals, and 5 schoolteachers. In particular, of the 33 parent participants, 19 were involved without their child and 14 had also their child interviewed; all siblings had also their brother or sister as patient interviewed. Therefore, 27 patients were interviewed and 19 were not but had their parents/siblings interviewed, resulting in 46 patients whose socio-demographic and clinical characteristics are described in Table 1. Among the patient participants, 15 (55.6%) were female, and 10 (37.04%) were between 18 and 24 years old. Overall, the most frequent diagnoses (*n* = 15, 32.61%) concerned the digestive system (e.g., Crohn's disease, ulcerative colitis), followed by the cardiovascular system (*n* = 7, 15.22%) such as myocarditis, and ventricular hypoplasia. Six patients (13.04%) were affected by a respiratory disease (e.g., cystic fibrosis), and another six (13.04%) were affected by a neurological/neuromuscular disease (e.g., epilepsy, spinal muscular atrophy 1). Moreover, 4 patients (8.70%) were affected by an autoimmune disease (e.g., rheumatoid arthritis, lupus erythematosus). Only three patients (6.52%) were affected by a dermatological disease (e.g., epidermolysis) and only two patients (4.35%) were affected by a nephrological disease (e.g., chronic kidney failure). The main list of diagnoses is available in Supplementary Table S2. For the totality of the patients, the median for the number of medications was 4 (IQR = 1–7.25). Medications were mainly taken via four routes: oral route (*n* = 39, 84.78%) followed by inhalational (*n* = 5, 10.87%), intravenous (*n* = 2, 4.35%), and ocular routes (*n* = 2; 4.35%). In terms of treatment, the use of devices (e.g., PEG, CVC, Port-a-cath) was the most frequent (*n* = 23, 49.98%) followed by electro-medical devices such as glucometers, spirometers, or defibrillation (*n* = 9, 19.55%). Three patients used non-pharmacological treatment (e.g., physiotherapy or PEP Mask) (*n* = 9, 19.55%).

**Table 1:** Characteristics of the patients (*n* = 46) and their families.

	Direct patient participants^a^ *N* = 27 (%)	Indirect patient participants^b^ *N* = 19 (%)	Total *N* = 46 (%)
Socio-demographic characteristics			
Age group			
0–2 years	0 (0)	6 (31.58)	6 (13.04)
3–5 years	0 (0)	2 (10.53)	2 (4.35)
6–10 years	6 (22.22)	7 (36.84)	13 (28.26)
11–13 years	4 (14.81)	1 (5.26)	5 (10.87)
14–17 years	7 (25.93)	3 (15.79)	10 (21.74)
18–24 years	10 (37.04)	0 (0)	10 (21.74)
Sex			
Female	15 (55.56)	9 (47.37)	24 (52.17)
Order of parentage			
Firstborn	14 (51.85)	11 (57.89)	25 (54.35)
Secondborn	11 (40.74)	8 (42.11)	19 (41.30)
Thirdborn	2 (7.41)	0 (0)	2 (4.35)
Region of residence			
Lazio	16 (59.26)	11 (57.89)	27 (58.70)
Other regions	11 (40.74)	8 (42.11)	19 (41.30)
Cohabitants of the patient			
Mother and father	2 (7.41)	6 (31.58)	8 (17.39)
Mother, father and siblings	21 (77.78)	11 (57.89)	32 (69.57)
Single parent and/or siblings	4 (14.81)	2 (10.53)	6 (13.04)
Attending school			
None or kindergarten	0 (0)	9 (47.37)	9 (19.57)
Elementary school	7 (25.93)	6 (31.58)	13 (28.26)
Middle school	4 (14.81)	1 (5.26)	5 (10.87)
High school or other	16 (59.26)	3 (15.79)	19 (41.30)
Mother's level of education			
Elementary or middle school	7 (25.93)	3 (15.79)	10 (21.74)
High school or university	20 (74.07)	16 (84.21)	36 (78.26)
Father's level of education			
Elementary or middle school	10 (37.04)	5 (26.32)	15 (32.61)
High school or university	17 (62.96)	14 (73.68)	31 (67.39)
Mother's job			
Unemployed	1 (3.70)	2 (10.53)	3 (6.52)
Stay-at-home mother	15 (55.56)	7 (36.84)	22 (47.83)
Worker	11 (40.74)	10 (52.63)	21 (45.65)
Father's job			
Worker	27 (100)	19 (100)	46 (100)
Patients’ clinical characteristics			
Diagnosis			
Digestive system	9 (33.33)	6 (31.58)	15 (32.61)
Cardiovascular	5 (18.52)	2 (10.53)	7 (15.22)
Respiratory	3 (11.11)	3 (15.79)	6 (13.04)
Neurological/neuromuscular	4 (14.81)	2 (10.53)	6 (13.04)
Autoimmune	3 (11.11)	1 (5.26)	4 (8.70)
Dermatological	2 (7.41)	1 (5.26)	3 (6.52)
Nephrological	1 (3.70)	1 (5.26)	2 (4.35)
Other diseases	0 (0)	3 (15.79)	3 (6.52)
Time (years) from diagnosis (mean, SD)	11.18 (6.12)	5.18 (4.09)	8.70 (6.10)
N. of medications (median, IQR)	4 (1–8)	5 (1–7)	4 (1–7.25)
Maximum intake of medications (per day) (median, IQR)	2 (1–3)	2 (1–3)	2 (1–3)
Routes of administration			
Oral route	24 (88.89)	15 (78.95)	39 (84.78)
Inhalational route	3 (11.11)	2 (10.53)	5 (10.87)
Topical route	1 (3.70)	0 (0)	1 (2.17)
Intravenous route	1 (3.70)	1 (5.26)	2 (4.35)
Intrathecal route	0 (0)	1 (5.26)	1 (2.17)
Ocular route	2 (7.41)	0 (0)	2 (4.35)
PEG	0 (0)	1 (5.26)	1 (2.17)
Type of treatment			
Pharmacological treatment	25 (92.59)	17 (89.47)	42 (91.30)
Non-pharmacological treatment	1 (3.70)	2 (10.53)	3 (6.52)
Devices	11 (40.74)	12 (63.16)	23 (50.00)
Electro-medical	5 (18.52)	4 (21.05)	9 (19.57)

SD, standard deviation; IQR, interquartile range.

^a^ Patients who actively participated in the study.

^b^ Patients whose only parents or siblings actively participated in the study.

Parents who directly participated in the study (*n* = 33), were mainly female (*n* = 27, 81.8%), their mean age was 43.0 years (SD = 8.7), and their level of education was high (see Supplementary Table S3). Fathers were all workers, whereas mothers were mainly stay-at-home mothers (*n* = 12, 44.4%) or unemployed (*n* = 3, 11.1%). Siblings were mainly female (*n* = 5, 83%), their mean age was 15.7 years (SD = 5.2), all attending school or university (*n* = 1, 16.7%), half of them were firstborns and the other half were secondborns.

Healthcare professionals were mainly female (*n* = 32, 97.0%) and their mean age was 47.2 years (SD = 5.2). Of these, 12 (36.4%) were physicians, 12 (36.4%) were nurses or pediatric nurses, 2 (6.0%) were psychologists and 7 (21.2%) were other healthcare providers (3 dieticians, 4 rehabilitation therapists) (see Supplementary Table S4). The majority (*n* = 17, 51.5%) of the healthcare professionals had more than 20 years of work experience. Schoolteachers were mainly female (*n* = 4, 80.0%), and their mean age was 53.4 years (SD = 3.4). In particular, 3 (60.0%) were teachers, one (20.0%) was a sports trainer, and one (20.2%) was a drama teacher. The majority (*n* = 3, 60.0%) of the schoolteachers had more than 20 years of work experience.

A total of 25 focus groups and 7 individual interviews were conducted. In particular, 7 focus groups and 3 interviews involved the patients, 8 focus groups and 3 interviews were conducted with the parents, 2 focus groups and one interview involved the siblings, 7 focus groups included the healthcare professionals, and one focus group included the schoolteachers (see Supplementary Table S5).

### 3.2. Emotional Text Mining results: factors

**Figure 1:**
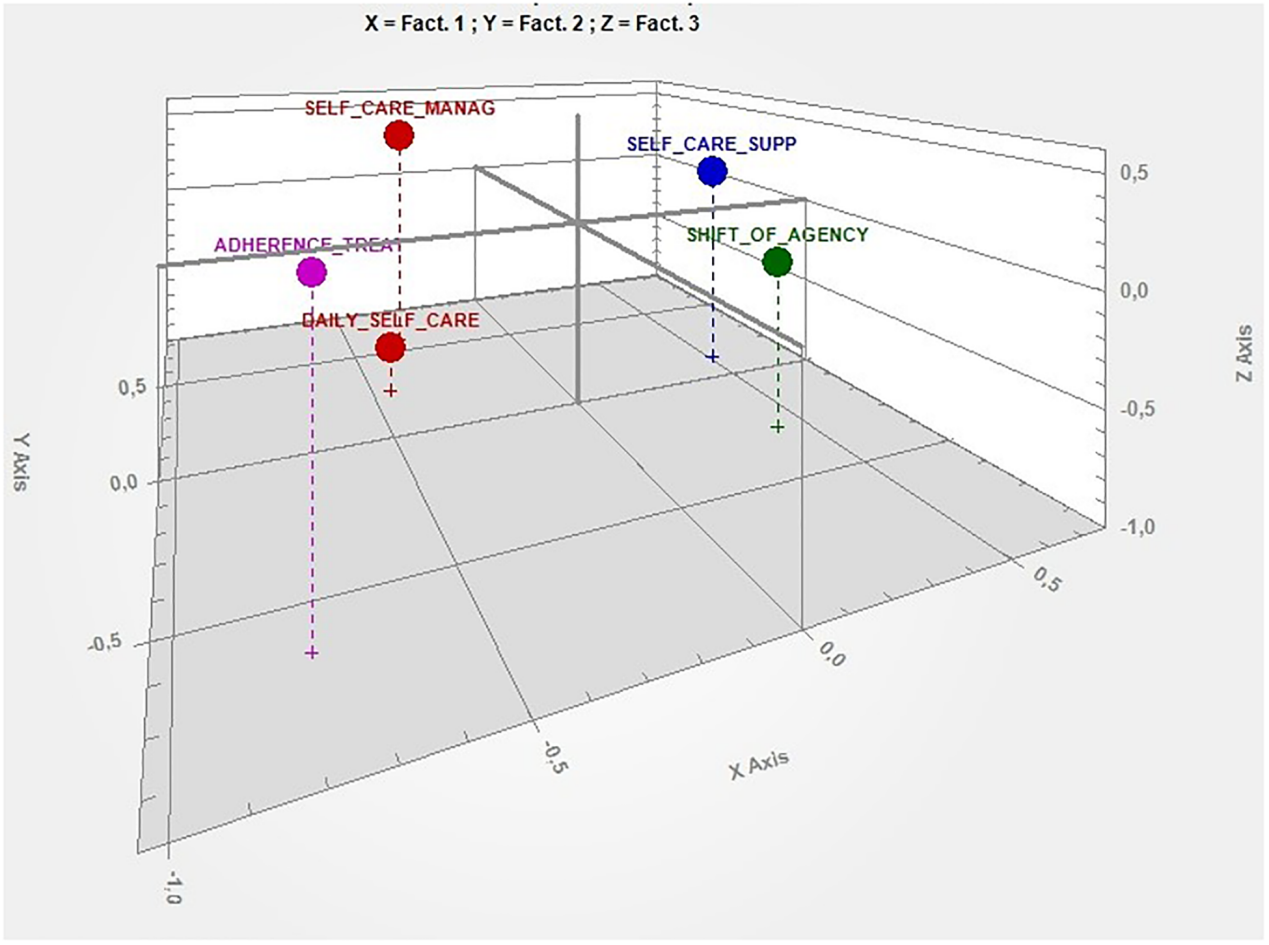
Factorial space produced by the first three factors and the location of the five clusters. X axis, self-care (− behaviours, +chronic condition); Y axis, external settings (− school, +hospital); Z Axis, family (− normal life, +management).

The results of the cluster analysis showed that the selected keywords (*n* = 364) enabled to classify 99.64% of the context units. The clustering validation measures showed that the optimal solution was five clusters (i.e., themes). Correspondence analysis detected four factors, and the explained inertia for each factor is reported in Table 2. Figure 1 shows the factorial space produced by the four factors and the location of the five clusters emerging from the focus groups and the interviews explaining 100% of the inertia.

**Table 2:** Results of the correspondence analysis.

Cluster	CU	CU%	Label	Factor 1	Factor 2	Factor 3	Factor 4
				Self-care	External settings	Family	Management
				38.7	23.7	19.8	17.7
Cluster 1	609	18.4%	Self-care management = Consulting/device	Behaviours	Hospital	Management of chronic conditions	Self-management
Cluster 2	894	27.0%	Shift of agency = School/parents/influencing factors	Chronic conditions	School		Self-management
Cluster 3	832	25.2%	Self-care support = Normal life/Multidisciplinary support	Chronic conditions			Professional support
Cluster 4	513	15.5%	Daily life self-care maintenance/monitoring	Behaviours	Hospital	Normal life	
Cluster 5	459	13.9%	Treatment adherence	Behaviours	School	Management of chronic conditions	

CU, context units classified in the cluster; CU%, percentage of the context units classified in the cluster. The second row shows the inertia explained by each factor.

As shown in Table 2, participants represented the self-care process via four main symbolic categories: Self-care, External settings, Family, and Management. The first factor highlighted the process of “self-care” as the impact of the “chronic condition” on patients' and families' lives (positive pole) and the “behaviors” (negative pole) that patients adopt during their activities of daily life.

The second factor focused on “external settings” where patients and families are engaged in self-care activities. In particular, the participants distinguished the “hospital” (positive pole), as the main landmark for patients and families to go and consult, from the “school” (negative pole), as the social setting where patients autonomously apply self-care behaviors such as taking medications.

The third factor highlighted the essential role of the “family” perceived as a comprehensive source of care over time. On the one hand, participants focused on the “management of chronic condition” (positive pole) perceived not only as the activities that patients and their families perform at home but also as their social and emotional response to the chronic condition. On the other hand, the family constituted the possibility to lead a “normal life” (negative pole) as much as possible.

Finally, the fourth factor focused on the “management” of the chronic condition both in a dependent and autonomous way. Participants distinguished “self-management” (positive pole), characterized by the importance of patients' empowerment in the experience of care during developmental age, and “professional support” (negative pole) provided by a multidisciplinary team both in the hospital setting and in the community. “Professional support” was considered fundamental to help patients cope with their chronic conditions.

### 3.3. Emotional Text Mining results: themes and sub-themes

Overall, five themes and seven sub-themes were identified from the data: (1) self-care management (Device management, Consulting behaviors); (2) shift of agency (Influencing factors, Parents, School); (3) self-care support (Normal life and personal development, Multidisciplinary support); (4) daily self-care maintenance/monitoring; (5) treatment adherence (Table 3). These themes and sub-themes reflected the process of self-care in children and young people with complex chronic conditions. This process is characterized by self-care behaviors such as maintenance (treatment adherence), monitoring, and management and evolves along with a continuum shift of agency. The process of self-care is influenced by multidisciplinary support.

**Table 3:** Clusters and verbatim quotations.

Theme	Subtheme	Verbatim quotation
Self-care management (1)	Device (1)	So, he reached his autonomy very early and also a lot of consciousness. For example, when we prepare the dressing, he knows that he must be still. Of course, sometimes he says “I get bored” so, you know, I put on cartoon videos or something to distract him for a while, but he knows he must stay still and we’ll end up soon. (Case 4, parent)
	Consulting (8)	Luckily, he never got sick here at home. Just once, he had this gastro-intestinal problem and so, we had to bring him to hospital. He had feces with blood, and they were stinky, and he had terrible abdominal pain so, we brought him to the hospital. Now, anyway, we set off a first aid system at home. (Case 2, parent)
Shift of agency (2)	Influencing factors (2)	Being able to interact with the care center…there are kids that when they got sick, there are parents that have to call the center. (Case 29, health professional)
	Parents (6)	Let me say one thing…interrupt their activities. Often, parents, literally a lot of parents, interrupt their lives. (Case 30, health professional)
	School (9)	I must say that the only difference I perceive is that I must respect her own time. She's got different timing in comparison with the other girls, and she needs a little more time to rest. But, after that, she responds nicely. (Case 31, schoolteacher)
Self-care support and education (3)	Normal life and personal development (3)	We do normal life. I mean, our little girl goes to school when she can, apparently, she gets to do sports. We do everything we can to give her a social life because it’s important to guarantee this. (Case 20, parent)
	Multidisciplinary support (7)	A psychological session… I guess this is fundamental for all the different stages of life that a patient with a chronic disease must face, linked to their disease. Also, we need a motivational aspect for the patient in self-care. This motivation goes hand in hand with the collaboration and the compliance, you know, to the adherence to self-care, in addition to the acceptance. (Case 29, health professional)
Daily life self-care maintenance/monitoring (4)		Sometimes we understand him from his mood when he's tired when he doesn't want to hear about anything at all, it's easy to understand, just from his mood […]. You can see from the start how it'll turn out, sometimes he wakes up in good spirits, sometimes in bad ones. (Case 9, sibling)
Treatment adherence (5)		Before going to school, in the morning, I wake up, have breakfast, take a pill, and then, I go back to sleep until half past seven when I have a second breakfast. I take others pills and then I go to school. When I come back home, I have to take other two important pills and also a diuretic. (Case 11, patient)

#### 3.3.1. Self-care management

Self-care management was described as the actions that patients and families perform when something fails, such as the appearance of symptoms or deteriorating clinical parameters. In particular, our qualitative analysis showed that self-care management included the sub-themes “device management” and “consulting behaviors”. Good device management was important for patients because a greater ability in managing devices could help them to improve their quality of life: *“And then, they told me “try to put this cap”, it's a sort of cork placed over the end of the tracheostomy, to close it. So, I put it every day and it was ok, I spoke, I chatted, I laughed” [CASE 03, patient]*. In addition, siblings played a crucial role in facilitating device management. Siblings acquired these abilities for a long time: *“I’m learning how to change these batteries and to be cautious too […] I'm learning that it's not so difficult to change them. Well, I also have the stress that the battery could die but for the rest, it's not very difficult to change it. They are instructing me, and I believe that I can do it very soon” [CASE 09, sibling]*.

Considering self-care management, consulting behaviors were found to be crucial to deal with critical events. In particular, consulting behaviors included calling health professionals to ask for help and suggestions, bringing their child to the pediatric hospitals, and activating an emergency service to visit the family at home promptly: *“I replaced it, then I called him because I live in Lecce [580 km from the hospital] and I had to call a physician of referral. […] I called asking “doctor, what should I do?” And he kindly called the pediatric surgery, and they helped me” [CASE 24, parent]*. In particular, nurse case managers might ensure the continuity of care, facilitating the ongoing management of the chronic conditions of patients and reassuring the parents: *“Sometimes he got sick, but we have this first aid anyway, and for us, Daniele is fundamental, truly. This figure, the VAD [Ventricular Assist Device] coordinator, helped us so much because we are literally safe when we are at home. I call him for everything so, in a moment, I feel safe” [CASE 02, parent]*.

#### 3.3.2. Shift of agency

Participants underlined the importance of a shift of agency. The shift of agency is the switch of responsibilities and performances of self-care behaviors from caregivers to patients themselves during developmental age. The qualitative analysis showed three sub-themes: influencing factors, parents, and school. The shift of agency started with the parents and is influenced by several factors. Concerning the children and young people, the shift of agency is manifest mainly in the school setting. One of the most important influencing factors was health-professional abilities to establish a good relationship with the family and to show empathy to improve the quality of care and understand patient's needs: *“And I must say that being able to understand the other point of view helps us to improve their relationship and the care path in some ways” [CASE 30, health professional]*.

Parents underlined the importance of receiving adequate education to manage their chronic conditions independently: *“So, considering the daily activities and treatments, and all the needs, in my opinion, the positive aspect is being able to do it all on your own. And having the means to learn something. This was extremely positive for me because at the beginning I asked for help to the nurses, the hospital, the center” [CASE 20, parent]*.

Parents are the main actors in providing care to their young children, but the constant provision of care may become burdensome for parents. Improving children's and young people's autonomy may result in a better quality of life for the parents: *“Of course, the parents’ burden goes down. That's not a small thing because the more the child is autonomous the more the burden for parents decreases in terms of autonomy” [CASE 26, health professional]*.

The health professionals explained the role of establishing a supportive network to help parents deal with their children. This network could include not only the family but also other caregivers and individuals such as neighbors: *“I know many parents, also ordinary people, who got home and, as my colleague was just saying, built a network, with grandparents, uncles, and aunts, with neighbors and parents who came and went from work to care for these kids” [CASE 28, health professional]*.

The schoolteachers affirmed that the main setting where the shift of agency occurred was the school. In particular, they underlined the importance of the role played by a figure who is familiar with the children and young people's condition and becomes a point of reference for other teachers: *“For me, it's a matter of mediation and coping with huge differences between children and teenagers. I noticed that for these kids it is useful to have a reference point in the school, someone who perfectly knows their condition” [CASE 31, health professional]*.

To better understand the child's difficulties and meet their needs, teachers expressed the need to guarantee continuity between hospitals and schools: *“We need a link between hospitals, therapists, and the team who have the kids in charge, with the teachers at the school attended by the kids because who*’*s going to see them?” [CASE 31, health professional]*.

#### 3.3.3. Self-care support

The analysis showed that self-care support includes two sub-themes: “normal life and personal development” and “multidisciplinary support”. Participants explained how the process of self-care should be facilitated through the support of health professionals to help patients achieve normal life and personal development. In particular, having a normal life was the main outcome reported by parents: *“Of course there are small precautions to be put in place, but the point is that she leads a normal life and yes, there are these little problems. For example, she sometimes worries about the loss of her hair due to tacrolimus but, you know, things go towards normality” [CASE 17, parent]*.

Also, health professionals were surprised and satisfied that many patients managed to lead a normal life despite their chronic conditions: *“So, the chronic conditions become part of themselves and so you find out that they are dating, they have a sexual life and they’re living a normal life despite everything” [CASE 30, health professional]*.

Participants stated how support should be multidisciplinary to be effective. Only multidisciplinary teams were considered able to manage complex chronic conditions. In the health professionals’ opinions, the teams should also include a psychologist: *“We talked and discussed the difficulties these patients could have, how we can help them and solve these problems. For example, with patients with spina bifida, we act like a team because they are patients with complex chronic conditions, so, a multidisciplinary team is necessary to deal with these patients periodically and there's a psychologist” [CASE 26, health professional]*.

Since patients with complex chronic conditions required care at home, health professionals emphasized the importance of providing self-care support at home. Nurses and physiotherapists were recognized as the main actors of self-care support at home: *“Nurses and physiotherapists [are important] to manage the patients at home because, you know, dealing with these conditions at home means that individuals, such as the parents of these kids with complex chronic conditions, have to do a lot of things that often are not linked to their profession, often they have completely different jobs” [CASE 29, health professional]*.

#### 3.3.4. Daily life self-care maintenance/monitoring

Daily life self-care included monitoring and maintenance behaviors aimed at promoting the stability of the complex chronic condition and checking clinical parameters and symptoms. Nutrition, according to patients, was an important daily life activity to stay healthy. Patients undertook to improve their diet to achieve better outcomes over time: *“First, I didn't eat much, but then I realized that I had to eat more so, every day I tried to eat a little more. I felt full but the next day I was hungrier” [CASE 03, patient]*.

Patients agreed that monitoring their health was based on detecting their sensations of feeling good or bad and their mood: *“And if you haven't been through the same situation you can't understand. So, we are all understanding each other. You realize you’re feeling good because you feel fine, more energetic, and shining. There's something you feel both when you’re ill or good, it's something you experience inside you” [CASE 11, patient]*.

#### 3.3.5. Treatment adherence

Parents recognized treatment adherence as a crucial part of their self-care. Taking medications was the most common self-care behavior reported by participants. This was performed alone or with the help of parents or other significant caregivers: *“He's able to take his medicines autonomously, for example, when he's all day out scouting, he knows that at lunchtime he has to take his medicines out from his little box and he takes them by himself, even though the scout leader knows he has to take it at that time. But we told him, don't say anything to remind him” [CASE 22, parent]*.

Patients reported that taking medications has an important impact on their daily routine. They adapted their activity of daily life based on the timing of the medications: *“Before going to school, in the morning, I wake up, I have breakfast, I take a pill and then, I go back to sleep until half past seven when I have a second breakfast. I take others pills and then I go to school. When I come back home, I have to take other two important pills and also a diuretic” [CASE 13, patient]*.

### 3.4. Participant's specificity related to the type of participants

Overall, there was a statistically significant difference in the participant's self-care perception based on the type of participants (*χ*^2^, *df* = 16; *p* < 0.001), particularly, between the family and health professionals or schoolteachers, as highlighted by the standardized residuals (Table 4). Parents and patients had a similar experience of self-care and focus mostly on self-care management, daily life self-care maintenance and monitoring, and treatment adherence, not attending to the shift of agency and self-care support. Siblings differed slightly from others, focusing only on daily life self-care maintenance and monitoring and treatment adherence, not attending to the shift of agency.

**Table 4:** Participant's specificity related to the type of participants.

	Self-care management	Shift of agency	Daily life self-care maintenance-monitoring	Self-care support and education	Treatment adherence
Parents (*n* = 33)	7.9^a^	−3.8^a^	2.0^a^	−6.4^a^	2.7^a^
Patients (*n* = 27)	3.2^a^	−7.2^a^	7.5^a^	−6.3^a^	6.9^a^
Siblings (*n* = 6)	−1.7	−2.1^a^	3.0^a^	−0.7	2.7^a^
Health professionals (*n* = 33)	−8.7^a^	7.6^a^	−8.4^a^	13.1^a^	−9.4^a^
Teachers (*n* = 5)	−4.0^a^	8.4^a^	−3.0^a^	−2.5^a^	−0.6

^a^ Standardized residual >│1.96│.

On the contrary, health professionals and schoolteachers focused mostly on the shift of agency, not attending to self-care management, and daily life self-care maintenance and monitoring. Health professionals also did not attend to treatment adherence, and they differed from teachers since they also addressed self-care support.

### 3.5. Participants’ specificity related to the patient age group

Overall, there was a statistically significant difference in the parents' and patients' self-care perception based on patients' age (*χ*^2^ parent, *df* = 20; *p* < 0.001; *χ*^2^ patient, *df* = 12; *p* < 0.001), particularly between the 6 months–3 year and the 18–24 year age groups, as highlighted by the standardized residuals (Table 5). No significant difference was found in siblings' self-care perception based on patient age. Parents and patients had different experiences of self-care based on patient age in most of the self-care aspects. Parents of children aged 6 months–3 years focused mostly on self-care management, not attending to shift of agency, self-care support, and treatment adherence. Parents of children aged 4–5 years focused mostly on treatment adherence, not attending to self-care support. Both patients aged 6–10 years and 11–13 years did not attend to shift of agency and self-care support, whereas for patients aged 18–24, these aspects were very relevant, in line also with their parents. Both parents and patients of the 18–24 year group did not attend to daily self-care maintenance/monitoring. Moreover, patients of the 18–24 year group focused on shift of agency, not attending to treatment adherence. Instead, the parents of children aged 18–24 years did not attend to self-care management.

**Table 5:** Parents’ and patients’ specificity related to the patient age group.

	Parents (*n* = 33)					Patients (*n* = 27)				
	Self-care management	Shift of agency	Daily life self-care maintenance-monitoring	Self-care support and education	Treatment adherence	Self-care management	Shift of agency	Daily life self-care maintenance-monitoring	Self-care support and education	Treatment adherence
6 months–3 years	4.80^a^	−1.96^a^	−2.55^a^	1.23	−2.82^a^					
4–5 years	−1.93	1.42	−2.11^a^	−0.27	3.22^a^					
6–10 years	−0.65	0.57	0.34	0.73	−0.88	0.38	−2.73^a^	−1.89	1.56	1.39
11–13 years	−0.78	0.61	0.25	−0.10	0.18	1.94	−2.09^a^	−3.80^a^	1.30	1.06
14–17 years	0.36	−1.27	0.11	−0.45	1.32	0.05	0.39	−1.78	0.33	0.63
18–24 years	−2.27^a^	0.41	5.16^a^	−2.30^a^	−0.15	−1.63	3.13^a^	5.05^a^	−2.20^a^	−2.11^a^

^a^ Standardized residual >│1.96│.

## 4. Discussion

This study explored self-care behaviors in children and young people with complex chronic conditions and investigated the main influencing factors of the self-care process associated with developmental age. To our knowledge, this is one of the first studies that explores self-care in children and young people with a wide range of complex chronic conditions and including diverse participant identities taking into account the perspectives of the key stakeholders, such as families and healthcare professionals. Our results shed light on the complexity of this phenomenon in this population and underline the common aspects across the various complex chronic conditions, such as the need to adhere to specific recommendations to preserve their health. Although the complexity of the chronic condition, developmental delay, and the limited life experiences may negatively impact on patients’ self-care capability in everyday life, this study showed that it is still possible to empower them to collaborate by performing self-care activities. Overall, our results are in line with the comprehensive model of self-care in children and young people with chronic conditions (18). Moreover, our study used an innovative approach for data analysis (Emotional Text Mining), which enabled us to detect the general topics and the cultural-symbolic categories influencing the self-care behaviors of the respondents.

Five themes were identified in this study summarizing the process of self-care during everyday life. The theme “self-care management” described what patients and families do in terms of “device management” and “consulting behaviors” when clinical conditions deteriorate.

The theme “shift of agency” showed the process of the transition of self-care responsibilities from parents to children and young people across different contexts of life and along developmental age, in line with the comprehensive model of self-care in children and young people with chronic conditions by Dall'Oglio et al. (18). Many factors were found to influence this process, such as a trusting relationship between health professionals and patients with their families. In particular, nurses should establish a genuine relationship with patients and families based on effective communication and compassion but also recognizing children's and young people's needs throughout the decision-making process (53–55). This relationship—nurses with patients and families—together with factors such as patient's developmental age/self-efficacy/socio-economic and cultural characteristics, might facilitate families in managing the disease and help them feel more empowered (56). The findings of this study also showed the key role played by parents as the main responsible persons for the self-care process. On one hand, parents become more knowledgeable, confident, skilled, and willing to care for their children's health over time, thereby parent activation increases and shapes their identity as caregivers (29, 57). On the other hand, the diagnosis and the management of the child's complex chronic conditions negatively affect parents' mental health increasing anxiety and depression (58, 59).

Health professionals should establish psychosocial interventions, such as promoting supportive programs based on cognitive behavioral therapy to lower parents' stress (60). Moreover, parents should receive practical and socio-economic support from families, friends, and the society to better manage the complex chronic conditions of their children. Among the contexts of life, the school was found to be the main setting where children and young people engage in self-care behavior independently. To facilitate this process, specific supportive figures, such as school nurses, could ensure their safety at school (61). In particular, it is crucial that school nurses are capable of managing the complex needs of students with chronic conditions, including devices, to establish a connection between the school and the hospital (62). Despite the engagement of teachers in promoting the social integration of the children and young people with complex chronic conditions, more should be done by other stakeholders such as school nurses to normalize their perception among other students in school contexts.

Another theme that was identified through this study was self-care support. Self-care support was considered fundamental for children and young people to conduct a normal life, despite the complex chronic condition, and to achieve personal development while their safety is ensured. This support should be provided by a multidisciplinary team. Participants emphasized that having a normal life means doing activities such physical exercises/practicing sports, meeting friends and having a social life, having a hobby, having a partner, and feeling integrated in the school context (63, 64). In order to help these children lead a normal life, it is important that people who live in the same contexts of life help them to engage in every normal activity, especially when they need more time to perform that specific activity, so that they may somehow achieve their developmental tasks.

The multidisciplinary support team played an important role in ensuring this process. Many healthcare professionals, such as pediatricians, nurses, and physiotherapists, are included in the multidisciplinary support network. Participants across all stakeholder groups considered the role of the psychologist as relevant in supporting the entire family from the time of diagnosis. Several studies showed that psychologists could improve the care provided and reduce the impact of complex chronic conditions on mental health (65). However, more interventions should be developed and implemented to help children and young people cope with their complex chronic conditions and anxiety (66, 67). Furthermore, peer support was considered helpful for children and young people. Since they were able to talk and become friends with their peers, the exchange of self-care experiences was facilitated and their engagement in self-care behaviors was increased (68). Health professionals should develop and reinforce peer support networks, as they are in the position to put children and young people and their families in touch, so that they may learn from each other.

Several studies have shown that caregivers and parents require psychological support (69, 70), but although several health promotion interventions have been developed, more extensive efforts should be made, especially for those families living far from their healthcare center. Parents should also receive support from a social network including friends and neighbors to reduce the impact of the condition on their lives and the consequent burden.

Another theme identified in this study was daily life self-care, which included maintenance behaviors to keep stability and monitoring behaviors to track parameters and symptoms. Nutrition, according to patients, was the most important daily life activity since they tended to modify their diet to improve their quality of life (71). In particular, for children and young people, activities correlated to lifestyle were simple everyday behaviors such as having breakfast, taking medication, going to school, resting at home, playing, sleeping, and for the adolescents having a social life. However, no preventive behaviors (such as hand washing) were explicitly reported. Probably, these preventive actions were considered an integral part of their usual behaviors, therefore, no particular attention was given to this aspect during the focus groups and interviews (72–74). Parents emphasized their attachment to their children during self-care monitoring activities that enabled them to recognize their health status. Some parents seemed to cultivate a symbiotic relationship with their children that did not evolve throughout the years towards the achievement of the child's independence. In order to help those parents place more confidence in the child's self-care abilities, health professionals should provide multidisciplinary support.

The last theme that was identified was “treatment adherence”. The daily life of children and young people with complex chronic conditions is usually marked by the need to take medications at specific times and modalities. A difficult treatment plan (i.e., with repeated doses at scheduled intervals) could reduce the ability of children and young people and their families to remain fully adherent (75). To facilitate adherence, it is important to negotiate the therapeutic regimen with patients and their families to make it suitable for their daily life (76).

Our findings confirmed that the self-care process is closely connected to the age of the involved individuals along with their development in the context of complex chronic conditions, as also found in cases of rare diseases (77). In particular, parents were very concerned about self-care management in early childhood, maybe because they were still learning and worried about what to do in case of unstable conditions (78), in line with the natural history of the disease (79). Instead, this aspect may be less relevant for parents of young people, probably because they have had more time to become experts in dealing with a complex chronic condition (80). Similarly, daily self-care maintenance/monitoring is not particularly considered by young people and their parents, probably because they no longer feel they should pay particular attention to this aspect anymore, as it is an intrinsic part of their life considering the necessities of their complex condition (81).

The aspect of treatment adherence becomes particularly relevant for parents when their children are 4–5 years old, and not earlier, maybe because at this age children's active collaboration on this task is necessary (82–84). This finding implies the need of involving younger children in education on treatment adherence. This education can be provided directly by healthcare professionals, with adequate communication tools, but also by helping the parents to relate with the child about this topic starting from the early years of life. On the one hand, treatment adherence does not seem to be relevant for patients who have reached the age of majority, who may feel mentally competent to decide for themselves. This finding may explain why poor adherence is often described as a big issue at this age (85, 86). On the other hand, the concept of shift of agency is a key topic for young people, as they seek to achieve autonomy and an independent life despite their chronic condition. Education on self-care behaviours could be anticipated at school and pre-adolescent age to facilitate shift of agency already in this period of life, even though patients do not attend to this aspect at that age. Moreover, it should be noted that both young people (18–24 years) and their parents are focused on self-care support, probably because they are facing challenges related to their own social inclusion at this age. Considering their special needs, society should understand how to promote the inclusion of these people across different contexts of adult life.

### 4.1. Implications for practice

Empowering patients and families to recognize and take actions in case of urgent need is important to manage the critical conditions at home and to reduce hospital readmission (87). Providing education to patients and families, on discharge but also during all the teachable moments, both at hospital and at home, might improve the patients' and families' skills and confidence in the management of devices (88, 89) and changing clinical conditions (90, 91). In addition, patients and parents should be educated to recognize when they really need to consult health professionals or go directly to hospital. Sutton et al. (92) underlined that a program including patient-care plans and care coordination, and 24-hour mobile-phone access could enhance families' abilities to manage their children's conditions at home (92). It is also important to underline the contribution of technology in providing self-care interventions and education for children with complex chronic conditions, to recognize symptoms and control exacerbations (93–95). Furthermore, for specific conditions, such as cardiac diseases, a nurse case manager like the VAD (Ventricular Assist Device) coordinator could improve the quality of follow up and increase the perceived safety of families at home (96). In addition, it is fundamental to extensively reinforce the provision of home care services for children and young people with complex chronic conditions, recognizing and addressing their special needs with competence (97).

### 4.2. Limitations

This study had some limitations. Although all the five types of participants were enrolled, we encountered some difficulties in the recruitment of fathers, siblings, and patients. Regarding fathers, it is possible that they were less involved because mothers were those mostly engaged in caring for the child while fathers were more focused on their own jobs. Regarding patients, we recruited fewer children and young people than expected because there were hard to identify by clinicians and after enrolment, some patients refused to participate just before beginning the focus group. In addition, some children and young people accepted to participate only if the parent was available, similarly to other qualitative studies (98–100). Future studies should identify specific strategies to promote the participation of fathers, siblings and children and young people. Moreover, siblings were expected to be three times as many but often parents were not willing to let siblings participate in the study to avoid being involved in emotionally demanding conversations. Secondly, health professionals were mostly women. This is consistent with the higher proportion of female workers within the hospital but could also be a selection bias due to a higher interest in self-care in females than males. Finally, this study merged data from focus groups and some individual interviews. This approach provided the possibility, for young children and unavailable parents, to participate in the study without losing their perspective on peculiar aspects of their own life.

## 5. Conclusions

This qualitative study emphasized the process of self-care in children and young people with complex chronic conditions underlying the importance of promoting self-care behaviors, such as maintenance (including treatment adherence), monitoring, and management. These self-care behaviours are performed with the important contribution of family members, who should receive ongoing daily support from health professionals and a social network (101). A key aspect of the process of self-care during developmental age is the shift of agency from parents to patients, intending to empower children and young people to achieve a normal life and personal development. Health professionals should therefore provide multidisciplinary self-care support over time by implementing educational interventions aimed at promoting self-care behaviors through training and simulation (102), thereby facilitating a shift of agency (103). For this purpose, it is important to foster children and young people's empowerment and active participation in managing their condition, along with the continuity of care, so as to limit adverse events and ensure patient safety (104).

Among the principal factors that could influence the self-care process, we should consider the different needs related to the specific developmental age and the evolution of the clinical condition over time. In particular, parents of younger children require greater support in the area of self-management concerning the unstable clinical conditions of their children. Another factor influencing self-care is social support offered by peers. Children and young people might benefit from peer support by establishing relationships with other children of similar developmental stages and/or complex conditions.

In conclusion, it could be interesting to explore self-care behaviors across cultures and different complex chronic conditions by performing gender-balanced focus groups. Future research could draw from our findings to develop a standard self-reporting tool to measure self-care behaviors in children and young people with complex chronic conditions, including all aspects of self-care (105). In this way, it will be possible to provide more patient and family-centered care and use this information to plan personalized care for children and young people with complex chronic conditions.

## Data Availability

The raw data supporting the conclusions of this article will be made available by the authors, without undue reservation.

## Self-care CYP Study Group

Bambino Gesù Children’s Hospital, IRCCS, Piazza Sant’Onofrio 4, 00165 Rome, Italy: Valentina Vanzi, Daniele Gargano, Alessandra Querciati, Marco Roberti, Manuel Pomponi, Anna Portanova, Tommaso Renzetti, Caterina Offidani.
