# Supplementary material for: Self-care in children and young people with complex chronic conditions: a qualitative study using Emotional Text Mining
Source: Front Pediatr. 2023 Jul 28;11:1170268. doi: 10.3389/fped.2023.1170268 (PMC10420086; doi:10.3389/fped.2023.1170268)
Supplement: Supplementary file 1 [file Datasheet1.pdf]

Supplementary Table 1

Self-care in children and young people with complex chronic conditions: A qualitative study using Emotional Text Mining

Giuseppina Spitaletta[$], Valentina Biagioli[$], Francesca Greco, Rachele Mascolo, Annachiara Liburdi, Giulia Manzi, Orsola Gawronski, Riccardo Ricci, Emanuela Tiozzo, Ercole Vellone, Teresa Grimaldi Capitello, Michele Salata, Massimiliano Raponi, Immacolata Dall'Oglio[*] and Self-care CYP Study Group

[$]These authors share first authorship

*** Correspondence:** Immacolata Dall'Oglio: immacolata.dalloglio@opbg.net

Supplementary Table 1. Interview guide of the focus groups and interviews

Participant	Questions
PARENTS	1. What do you do to take care of your children? What kinds of attitudes, actions, activities do you usually practice? Can you tell me about your experience also with examples, if you like?<br>• 1.1 How do you know that your children are doing well? And what do you do if something is going wrong?<br>2. What do your children do or should do to take care of themselves? Can you tell me about your experience also with examples, if you like?<br>• 2.1 How do your children know they are fine? And what do they do if there's something wrong?<br>3. The perceived problems are various but I was wondering if you've ever thought about possible solutions and, if so, which?
PATIENTS (18-24)	1. What do you do to take care of yourselves? What kinds of attitudes, actions, activities do you usually practice? Can you tell me about your experience also with examples, if you like?<br>• 1.1 How do you know you're fine? And what do you do if you are not ok?<br>2. What do your parents do to take care of you?<br>• 2.1 How do your parents know you are fine? And what do they do if you are not ok?<br>3. The perceived problems are various but I was wondering if you've ever thought about possible solutions and, if so, what?
PATIENTS (13-17)	1. What do you do to take care of yourselves? Can you tell me about your experience also with examples, if you like?<br>• 1.1 How do you know you're fine? And what do you do if you are not ok?

2. Thinking about your daily life, what do your parents do to help you take care of yourselves?

   - 2.1 Did they educate you to do something by yourselves?

3. The problems you told me are various but I was wondering if you've ever thought about possible solutions and, if so, what?

PATIENTS (6-12)

1. What do you do every day to feel ok and healthy? Can you tell me about your experience also with examples, if you like?

- 1.1 How do you know you're fine? And what do you do if you are not ok?

2. What do your parents do to help you feel ok?

- 2.1 How do your parents know you are fine? And what do they do if you are not ok?

3. You told us that not everything goes well, I was wondering if you've ever thought about possible solutions to make things better?

SIBLINGS

(13-24)

1. Thinking about your daily life, what do your siblings do to take care of themselves? Can you tell me about your experience as siblings also with examples, if you like?

   - 1.1 How do your siblings know they are fine? And what do they do if they are not ok?

2. What do you do to help your siblings take care of themselves?

   - 2.1 How do you know they're fine? And what do you do if they are not ok?

3. The problems you told me are various but I was wondering if you've ever thought about possible solutions and, if so, which?

SIBLINGS (6-12)

1. Thinking about your siblings, what do your siblings do to take care of themselves daily? Can you think about some examples?

   - 1.1 How do your siblings know they are fine? And what do they do if they are not ok?

2. How did you help them?

   - 2.1 How do you know they're fine? And what do you do if they are not ok?

	3. You told us that not everything goes well, I was wondering if you've ever thought about possible solutions to make things better?
HEALTHCARE PROFESSIONALS	1. Thinking about the daily life of children/young adults with chronic conditions, what actions do they practice routinely to take care of themselves? Can you tell me about your experience? Can you think about some examples?<br>• 1.1 What actions do the children/young adults practice to check their health condition? And which actions do they put in place when they see changes in health?<br>2. What are the activities that parents/caregivers put in place to help their children take care of themselves?<br>• 2.1 What are the activities that parents put in place to check the health condition of their children? And which actions do they put in place when they see changes in health?<br>3. The perceived problems are various but I was wondering if you've ever thought about possible solutions and, if so, which?
OPERATORS OF CONTEXTS OF LIFE	1. Thinking about the health needs of children/young adults with chronic conditions, what do you think they could do to take care of themselves? Can you tell me about your experience with this category of children/young adults? Can you think about some examples?<br>• 1.1 How do the children/young adults know they are fine? And what do they do if they are not ok?<br>2. What could you do to help children/young adults take care of themselves? Can you tell me about your experiences? Can you think about some examples?
