# Supplementary material for: Self-care in children and young people with complex chronic conditions: a qualitative study using Emotional Text Mining
Source: Front Pediatr. 2023 Jul 28;11:1170268. doi: 10.3389/fped.2023.1170268 (PMC10420086; doi:10.3389/fped.2023.1170268)
Supplement: Supplementary file 2 [file Datasheet2.pdf]

*Supplementary Table 2*

# Self-care in children and young people with complex chronic conditions: A qualitative study using Emotional Text Mining

**Giuseppina Spitaletta[$], Valentina Biagioli[$], Francesca Greco, Rachele Mascolo, Annachiara Liburdi, Giulia Manzi, Orsola Gawronski, Riccardo Ricci, Emanuela Tiozzo, Ercole Vellone, Teresa Grimaldi Capitello, Michele Salata, Massimiliano Raponi, Immacolata Dall'Oglio[*] and Self-care CYP Study Group**

[$]These authors share first authorship

*** Correspondence:** Immacolata Dall'Oglio: immacolata.dalloglio@opbg.net

**Supplementary Table 2**. Type of diagnosis (n=46)

| TYPE OF DIAGNOSIS | DIAGNOSIS | N |
|---|---|---|
| **Autoimmune diseases** | Rheumatoid arthritis | 1 |
| | Hyper IgE Syndrome | 1 |
| | Lupus erythematosus | 1 |
| | SAVI syndrome | 1 |
| **Cardiovascular disease** | Dilated cardiomyopathy | 1 |
| | Anomalous coronary artery | 1 |
| | Ventricular hypoplasia | 1 |
| | Myocarditis | 2 |
| | Transposition of the great vessels | 1 |
| | Truncus arteriosus | 1 |
| **Dermatological diseases** | Dystrophic epidermolysis bullosa | 2 |
| | Epidermolysis bullosa | 1 |
| **Diseases of the digestive** | Biliary atresia | 3 |

| | | |
|---|---|---|
| **system** | Esophageal atresia | 1 |
| | Liver cirrhosis | 1 |
| | Sclerosing cholangitis | 1 |
| | Outcomes of hepatocarcinoma | 1 |
| | Intestinal failure | 1 |
| | Short gut syndrome | 3 |
| | Crohn's disease | 1 |
| | Chronic intestinal pseudo-obstruction | 1 |
| | Ulcerative colitis | 2 |
| **Nephrological diseases** | Chronic renal failure | 1 |
| | Steroid-resistant nephrotic syndrome | 1 |
| **Neurological /** | Duchenne muscular dystrophy | 1 |
| **Neuromuscular disease** | Proximal spinal muscular atrophy type 1 | 1 |
| | Epilepsy | 1 |
| | Spina bifida | 3 |
| **Respiratory diseases** | Laryngo-tracheo-esophageal cleft | 1 |
| | Cystic fibrosis | 5 |
| **Other diseases** | Cystinosis | 2 |
| | ROHHADNET Syndrome | 1 |

frontiers
