# Supplementary material for: Self-care in children and young people with complex chronic conditions: a qualitative study using Emotional Text Mining
Source: Front Pediatr. 2023 Jul 28;11:1170268. doi: 10.3389/fped.2023.1170268 (PMC10420086; doi:10.3389/fped.2023.1170268)
Supplement: Supplementary file 3 [file Datasheet3.pdf]

Supplementary Table 3

Self-care in children and young people with complex chronic conditions: A qualitative study using Emotional Text Mining

Giuseppina Spitaletta[$], Valentina Biagioli[$], Francesca Greco, Rachele Mascolo, Annachiara Liburdi, Giulia Manzi, Orsola Gawronski, Riccardo Ricci, Emanuela Tiozzo, Ercole Vellone, Teresa Grimaldi Capitello, Michele Salata, Massimiliano Raponi, Immacolata Dall'Oglio[*] and Self-care CYP Study Group

[$]These authors share first authorship

*** Correspondence:** Immacolata Dall'Oglio: immacolata.dalloglio@opbg.net

Supplementary Table 3. Characteristics of the parents actively participating in the study (n=33)

	**N (%)**
**Sex**	
Male	6 (18.18)
Female	27 (81.82)
**Average age (mean; SD)**	43.03 (8.71)
**Nationality**	
Italian	31 (93.94)
Romanian	2 (6.06)
**Kinship**	
Mother	27 (81.82)
Father	6 (18.18)
**Number of family members**	
≤3	9 (27.27)

>3	24 (72.73)
**Region of residence**	
Lazio	21 (63.64)
Others	12 (36.36)
**Level of education**	
Elementary school	1 (3.03)
Middle school	5 (15.15)
High school	17 (51.52)
University	10 (30.30)

SD= Standard Deviation
