# Supplementary material for: Self-care in children and young people with complex chronic conditions: a qualitative study using Emotional Text Mining
Source: Front Pediatr. 2023 Jul 28;11:1170268. doi: 10.3389/fped.2023.1170268 (PMC10420086; doi:10.3389/fped.2023.1170268)
Supplement: Supplementary file 4 [file Datasheet4.pdf]

Supplementary Table 4

Self-care in children and young people with complex chronic conditions: A qualitative study using Emotional Text Mining

Giuseppina Spitaletta[$], Valentina Biagioli[$], Francesca Greco, Rachele Mascolo, Annachiara Liburdi, Giulia Manzi, Orsola Gawronski, Riccardo Ricci, Emanuela Tiozzo, Ercole Vellone, Teresa Grimaldi Capitello, Michele Salata, Massimiliano Raponi, Immacolata Dall'Oglio[*] and Self-care CYP Study Group

[$]These authors share first authorship

*** Correspondence:** Immacolata Dall'Oglio: immacolata.dalloglio@opbg.net

Supplementary Table 4. Characteristics of healthcare professionals (n = 33)

	**N (%)**
**Sex**	
Male	1 (3.03)
Female	32 (96.97)
**Age (mean, SD)**	47.24 (11.01)
**Nationality:** Italian	33 (100)
**Children**	
Yes	20 (60.60)
No	13 (39.39)
**Professional qualification**	
Nurse	1 (3.03)
Pediatric Nurse	12 (36.36)
Physician	11 (33.33)

Rehabilitation Therapist	4 (12.12)
Psychologist	2 (6.06)
Dieticians	3 (9.09)
**Level of education**	
High School	3 (9.09)
Bachelor’s Degree	12 (36.36)
Master’s Degree	6 (18.18)
Medical Specialty	11 (33.33)
Regional Advance Course	1 (3.03)
**Years of experience**	
$1 \le x \le 10$	8 (24.24)
$11 \le x \le 20$	8 (24.24)
>20	17 (51.52)

SD= Standard Deviation
