# Supplementary material for: Self-care in children and young people with complex chronic conditions: a qualitative study using Emotional Text Mining
Source: Front Pediatr. 2023 Jul 28;11:1170268. doi: 10.3389/fped.2023.1170268 (PMC10420086; doi:10.3389/fped.2023.1170268)
Supplement: Supplementary file 5 [file Datasheet5.pdf]

Supplementary Table 5

Self-care in children and young people with complex chronic conditions: A qualitative study using Emotional Text Mining

Giuseppina Spitaletta[$], Valentina Biagioli[$], Francesca Greco, Rachele Mascolo, Annachiara Liburdi, Giulia Manzi, Orsola Gawronski, Riccardo Ricci, Emanuela Tiozzo, Ercole Vellone, Teresa Grimaldi Capitello, Michele Salata, Massimiliano Raponi, Immacolata Dall'Oglio[*] and Self-care CYP Study Group

[$]These authors share first authorship

*** Correspondence:** Immacolata Dall'Oglio: immacolata.dalloglio@opbg.net

Supplementary Table 5. Characteristics of focus groups and interviews

**Case**	**Interview /Focus group**	**Participants** (Type)	**Participants** (N)	**Gender**	**Participants Age/Mean Age** (Range)	**Patients Mean Age** (Range)	**Duration** (Min)	**CL1** (Tok)	**CL2** (Tok)	**CL3** (Tok)	**CL4** (Tok)	**CL5** (Tok)
1	I	P	1	M	9.6	9.6	32:18	1	0	0	13	12
2	I	P	1	M	10.4	10.4	46:27	34	4	9	20	23
3	I	P	1	M	11.5	11.5	52:28	35	0	1	36	16
4	I	PR	1	M	34	7.7	51:27	31	22	19	29	11
5	I	PR	1	F	38	5.3	24:28	13	10	3	3	14
6	I	PR	1	F	22	1.6	22:06	10	4	1	7	6
7	I	S	1	F	17	18.4	24:21	8	7	10	20	8
8	F	S	2	2F	21.5 (20-23)	20.4 (18-22)	37:40	7	21	25	7	11
9	F	S	3	2F 1M	11 (9-12)	12.3 (8-19)	01:14:22	16	17	17	26	28
10	F	P	2	2F	17.9 (17-18)	17.9 (17-18)	36:39	11	10	10	12	16
11	F	P	5	3F 2M	19.1 (18-21)	19.1 (18-21)	01:21:15	40	32	34	26	27
12	F	P	4	4F	20.6 (18-22)	20.6 (18-22)	41:02	5	15	26	21	9
13	F	P	4	2F 2M	15.8 (14-17)	15.8 (14-17)	35:23	13	7	4	6	15
14	F	P	2	2M	8.5 (15-16)	8.5 (15-16)	41:26	14	9	4	26	15
15	F	P	3	3M	12.2 (10-13)	12.2 (10-13)	28:57	9	9	1	9	23
16	F	P	4	4F	9.5 (8-10)	9.5 (8-10)	36:25	2	3	2	16	8
17	F	PR	4	3F 1M	52.7 (40-59)	18.1 (17-18)	01:01:19	16	23	36	8	16
18	F	PR	6	6F	45 (39-47)	15.8 (15-16)	01:18:06	39	21	21	21	28
19	F	PR	4	4F	48 (44-53)	12.1 (11-13)	01:13:00	31	30	21	22	22
20	F	PR	4	4F	41.25 (31-46)	8.5 (5-10)	01:21:47	31	33	26	29	23
21	F	PR	2	2F	33 (31-35)	7.1 (6-8)	57:36	32	23	14	24	21

22	F	PR	3	3M	49.6 (44-57)	9.4 (8-10)	52:44	28	25	16	6	14
23	F	PR	3	2F 1M	40.6 (30-52)	4.3 (1-7)	01:08:31	22	35	13	26	32
24	F	PR	4	3F 1M	37.5 (32-42)	1.4 (7 months-2)	01:23:24	79	24	15	34	10
25	F	HP	5	5F	56.8 (50-63)	-	01:41:24	8	61	61	8	13
26	F	HP	3	3F	40.6 (30-59)	-	01:15:13	8	69	42	4	2
27	F	HP	6	6F	42.3 (31-59)	-	01:07:19	12	61	42	11	3
28	F	HP	4	4F	54.2 (47-57)	-	01:09:05	18	47	78	10	4
29	F	HP	6	5F 1M	43.5 (30-58)	-	01:22:43	5	58	96	3	5
30	F	HP	4	4F	47 (32-57)	-	01:17:49	13	62	69	9	3
31	F	T	5	4F 1M	53.4 (52-59)	-	01:13:13	6	91	21	8	17
32	F	HP	5	5F	46.6 (33-64)	-	01:24:42	11	61	96	13	4

CL = Cluster; I = Interview; F = Focus; P = Patient; PR = Parents; S = Siblings, HP = Healthcare professionals, T = Teacher, Tok=number of Token, a single entity that is building blocks for sentence or paragraph
